# Microbial signatures in human periodontal disease: a metatranscriptome meta-analysis

**DOI:** 10.3389/fmicb.2024.1383404

**Published:** 2024-04-09

**Authors:** Armen Ovsepian, Filippos S. Kardaras, Anargyros Skoulakis, Artemis G. Hatzigeorgiou

**Affiliations:** ^1^DIANA-Lab, Department of Computer Science and Biomedical Informatics, University of Thessaly, Lamia, Greece; ^2^Department of Microbiology, Hellenic Pasteur Institute, Athens, Greece

**Keywords:** periodontitis, metatranscriptomics, meta-analysis, microbial network analysis, microbiome, core-microbiome, oral microbiome, dysbiosis

## Abstract

The characterization of oral microbial communities and their functional potential has been shaped by metagenomics and metatranscriptomics studies. Here, a meta-analysis of four geographically and technically diverse oral shotgun metatranscriptomics studies of human periodontitis was performed. In total, 54 subgingival plaque samples, 27 healthy and 27 periodontitis, were analyzed. The core microbiota of the healthy and periodontitis group encompassed 40 and 80 species, respectively, with 38 species being common to both microbiota. The differential abundance analysis identified 23 genera and 26 species, that were more abundant in periodontitis. Our results not only validated previously reported genera and species associated with periodontitis with heightened statistical significance, but also elucidated additional genera and species that were overlooked in the individual studies. Functional analysis revealed a significant up-regulation in the transcription of 50 gene families (UniRef-90) associated with transmembrane transport and secretion, amino acid metabolism, surface protein and flagella synthesis, energy metabolism, and DNA supercoiling in periodontitis samples. Notably, the overwhelming majority of the identified gene families did not exhibit differential abundance when examined across individual datasets. Additionally, 4 bacterial virulence factor genes, including TonB dependent receptor from *P. gingivalis*, surface antigen BspA from *T. forsynthia*, and adhesin A (PsaA) and Type I glyceraldehyde-3-phosphate dehydrogenase (GAPDH) from the *Streptococcus* genus, were also found to be significantly more transcribed in periodontitis group. Microbial co-occurrence analysis demonstrated that the periodontitis microbial network was less dense compared to the healthy network, but it contained more positive correlations between the species. Furthermore, there were discernible disparities in the patterns of interconnections between the species in the two networks, denoting the rewiring of the whole microbial network during the transition to the disease state. In summary, our meta-analysis has provided robust insights into the oral active microbiome and transcriptome in both health and disease.

## 1 Introduction

The oral microbiome is one of the most complex and diverse microbial communities in the human body, harboring hundreds of species (Huttenhower et al., 2012). The oral sub-habitats include the buccal mucosa, the tongue dorsum and the hard structures of the teeth, which are comprised by those above (supragingival) and below (subgingival) the gingival margin (Xu et al., 2015). Despite the close proximity of oral sub-habitats, the distinct ecological conditions that prevail in each of them create special niches and contain markedly different microbial communities (Hoare et al., 2017).

The microorganisms of the oral microbiome are entangled in a wide range of interspecies interactions, which include synergistic, signaling, or antagonistic interactions and polymicrobial biofilm formation (Jakubovics et al., 2000; Diaz and Valm, 2020; Radaic and Kapila, 2021). Qualitative and/or quantitative shifts of the oral microbiome lead to microecological dysbiosis, an imbalance responsible for the development of periodontitis, which is a chronic oral inflammatory disease that progressively destroys the supporting periodontal tissues (Jakubovics et al., 2000; Huang et al., 2021; Morillo-Lopez et al., 2022). Dental plaque biofilms play an essential role in the initiation and progression of periodontitis (Jakubovics et al., 2000; Morillo-Lopez et al., 2022). The prevalence of periodontitis is high, with approximately 10% of the global population being affected by severe periodontitis (Frencken et al., 2017).

Metagenomics and, more recently, metatranscriptomics studies have been employed to better understand periodontitis and unravel the molecular mechanisms involved in the disease (Huang et al., 2021). Metagenomics offers information on genes or microorganisms that are or have been present in the community. Metatranscriptomics could provide a broader perspective than metagenomics, as it can reveal details about transcriptionally active populations, functional characteristics of complex microbial communities in health or disease, and elucidate potential intervention targets (Huang et al., 2021; Ojala et al., 2023). Additionally, total RNA-Seq has been shown to be more accurate in microbial identification accuracy than metagenomics, at equal sequencing depths and even at sequencing depths almost one order of magnitude lower than those of metagenomics (Hempel et al., 2022). While metagenomics and metatranscriptomics have revolutionized our understanding of microbial communities and their functional potential, a number of important questions remain to be addressed. For instance, how do alterations in the oral microbial composition intricately contribute to the pathogenesis of periodontitis? Could the disease progression be attributed to the structural shifts of the entire microbial network, or to distinct components within the oral microbiota? Which are the most important microbial interactions during dental plaque development and disease progression?

The results of individual metagenomics and metatranscriptomics studies are often insufficient to provide confident answers, as they are not consistently reproducible (Armour et al., 2019; Huang et al., 2021). This situation is further impaired by the lack of standards in metagenomics and metatranscriptomics data generation and processing (Costea et al., 2017) and the relatively low number of samples used. In this meta-analysis, we examine existing metatranscriptome datasets to identify active signature microbial species associated with periodontitis and shed light on the functional characteristics of the microbial community of dental plaque.

## 2 Materials and methods

### 2.1 Study inclusion and data acquisition

PubMed was used to retrieve studies containing oral shotgun metatranscriptome data from subgingival samples in both patients with periodontitis and healthy individuals. The search terms were “periodontitis” AND “metatranscriptome,” and results were collected up until June 1, 2023. In total, 48 articles were identified, 11 of which were review articles and were excluded from the analysis. An examination of the references cited in these review articles led to the addition of 1 more article (Duran-Pinedo et al., 2014) to our list. All search hits, except review articles, along with the additional study dataset, and the justification for exclusion or inclusion in our study are available in Supplementary Table S1. Ultimately, the datasets from four articles were included in the meta-analysis and periodontitis samples from all studies had pocket depth (PD) ≥ 4.0 mm and clinical attachment loss (CAL) > 3 mm. The data were downloaded from either the National Library of Medicine (NCBI) Sequence Read Archive (SRA) or the Human Oral Microbiome Database (HOMD). The BioProject accession numbers were PRJNA678453 (Belstrøm et al., 2021) and PRJNA221620 (Jorth et al., 2014). The data from HOMD had the submission numbers 20141024 (Yost et al., 2015) and 20130522 (Duran-Pinedo et al., 2014). The Belstrøm et al. (2021) data was produced in an Illumina HiSeq2500 platform at a read length of 2 × 100 bp, the Yost et al. (2015) and Duran-Pinedo et al. (2014) data in an Illumina MiSeq v2 at read lengths of 2 × 75 and 2 × 150 bp, respectively, and the Jorth et al. (2014) data in an Illumina HiSeq2000 with 50-bp single-end reads length.

### 2.2 Data preprocessing

The lllumina 3′ adapters (5′AGATCGGAAGAGCACACGTCTGAACTCCAGTCA3′ from read 1, 5′AGATCGGAAGAGCGTCGTGTAGGGAAAGAGTGT3′ from read 2 - if applicable -, and 5′CTGTCTCTTATACACATCT3′ from both read 1 and read 2) were removed from the sequencing reads by Atropos (Didion et al., 2017). A quality cutoff of 20 was used to trim low-quality 3′ ends from reads before adapter removal. Then, rRNA reads were eliminated from the data by using sortmeRNA (Kopylova et al., 2012) version 2.1 and the default parameters. Human reads were removed from the data by aligning reads to the human genome GRCh38, NCBI RefSeq assembly GCF_000001405.40. For the alignment, HISAT2 (Kim et al., 2015) v.2.2.1 was employed and pairs that did not align concordantly were considered reads of microbial origin.

### 2.3 Taxonomic profiling, functional analysis, and virulence factor genes detection

Taxonomic profiling of metatranscriptomes was performed with AGAMEMNON (Skoufos et al., 2022). For the creation of the database, all the reference and representative bacterial genomes (4,264 in total), all complete archaeal (492) and viral (11,284) genomes, available as of 31st of January 2023, and all available fungal transcriptomes (489), as of 24th of March 2023, were retrieved from the NCBI RefSeq database (O’Leary et al., 2016).

For the identification of bacterial virulence factors, the bacterial virulence factor full DNA dataset (VFDB) (Liu et al., 2022) was downloaded on the 13th of February, 2023. Then, preprocessed reads were mapped to the database with Bowtie 2 (Langmead and Salzberg, 2013). From the generated SAM files, samtools were used to extract the virulence factor reference names and the number of reads that were uniquely mapped to each of them.

HUMAnN 3 (Beghini et al., 2021) was used for functional analysis. Briefly, preprocessed reads were mapped using Bowtie2 to the full chocophlan pangenomes (version: v201901_v31). Unaligned reads were then blasted against UniRef90 (version: uniref90_201901b) using DIAMOND (Buchfink et al., 2015). Counts were assigned to gene families and were normalized for length (reads per kilobase).

### 2.4 Data handling

α-diversity and β-diversity were assessed using the Shannon diversity index and Bray–Curtis dissimilarity, respectively. These calculations were performed on data that had been normalized using the Cumulative Sum Score (CSS) method. The CSS normalization was executed in R, utilizing the “metagenomeSeq” package (version 1.40.0). To evaluate the significance of differences in Shannon diversities between the healthy group and the periodontitis group, a blocked Wilcoxon test was employed. This test was implemented using the R “coin” package (Hothorn et al., 2006). Principal Coordinates Analysis (PCoA) was performed on the Bray–Curtis dissimilarity matrix to visualize and compare the microbial communities across samples and groups.

The core microbiota was identified based on two key criteria: species occurrence within the study group and in-sample relative abundance. Specifically, a species was included in the core microbiota if it met the following conditions: (a) the species had to be present in at least 80% of the samples, reflecting a high degree of occurrence within the study group, and (b) the species had to be within the top 25% of in-sample species relative abundances. These stringent criteria ensure that the core microbiota comprises species that are both prevalent across samples and abundant within individual samples.

For differential abundance analysis, MaAsLin2 (Mallick et al., 2021) was used. For microbial species, gene families and virulence factors, the study was set as a random effect and a negative binomial regression model was applied to the CSS normalized counts data. The prevalence threshold (min_prevalence parameter) for the microbial species was set at 0.5 (i.e., 50% prevalence), while for gene families and the virulence factor genes at 0.25. The Benjamini-Hochberg (BH) method was used for multiple hypothesis correction and estimation of *q*-values (FDR values).

The bacterial co-occurrence networks were created and analyzed using the R package “NetComi” (Peschel et al., 2021). Separate networks were created for the periodontitis group and the healthy controls with the Sparse InversE Covariance estimation for Ecological Association and Statistical Inference (SpiecEasi) algorithm (Kurtz et al., 2015), using the top 100 bacterial species with the highest median relative abundance per group; neighborhood selection method “mb” was used for computing the sparse inverse covariance matrix and the optimal lambda values were identified by setting “nlambda = 10” and “lambda.min.ratio = 0.1.” For the clustering of bacterial species, the algorithm “cluster_fast_greedy” was used. As hub species of the co-occurrence networks were defined the species with degree and betweenness greater than the 85th quartiles.

## 3 Results

### 3.1 Studies inclusion

**Figure 1:**
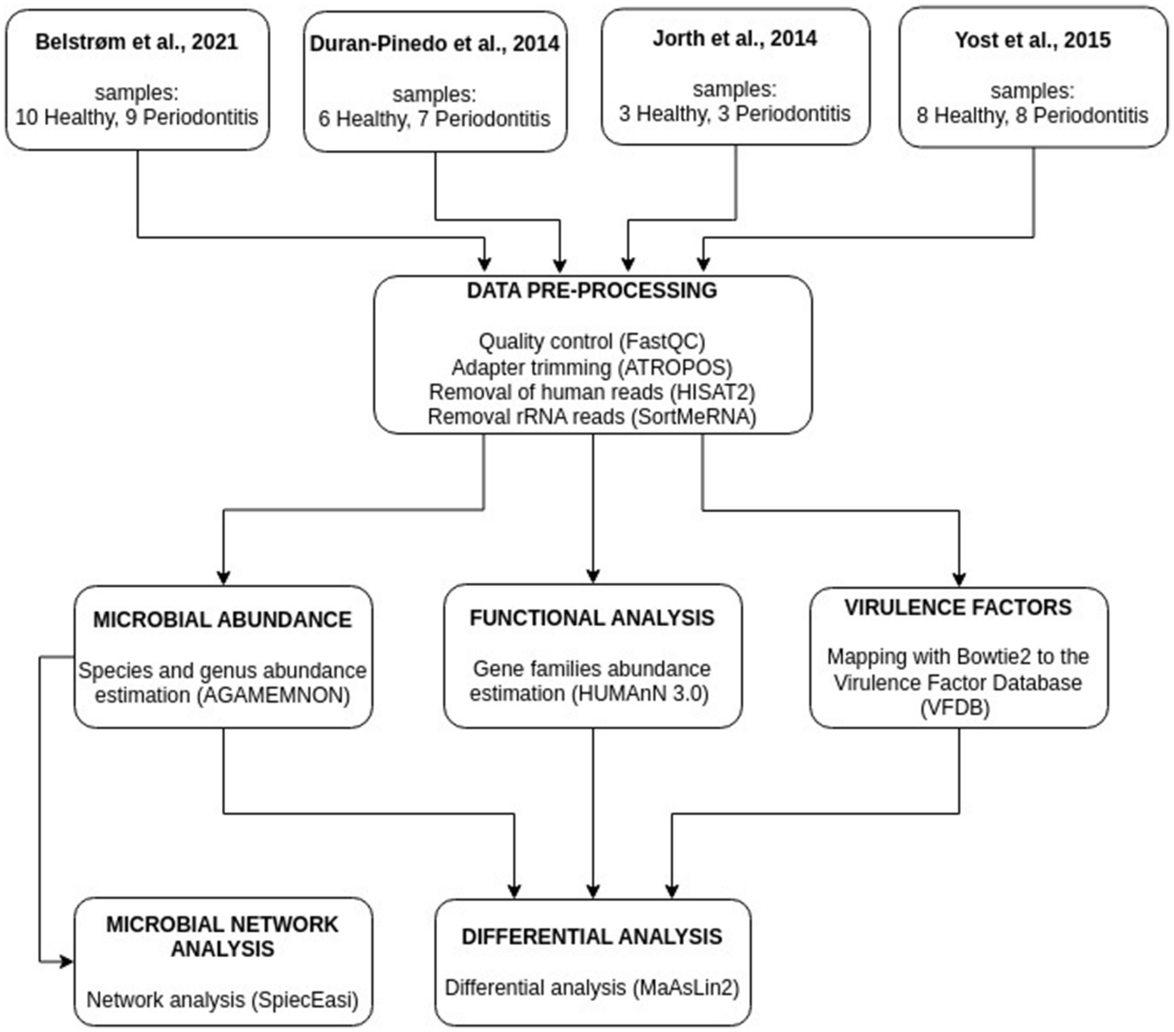
Illustration of the meta-analysis workflow integrating metatranscriptomics data from four studies. Abundances of microbial species, gene families, and virulence factor genes were estimated. Networks based on microbial species abundances were constructed for the healthy and the periodontitis groups.

In this meta-analysis, four studies containing human oral metatranscriptome data with healthy and periodontitis subgingival plaque samples, were used. All studies, as well as the type and the number of samples used, can be found in Table 1. In total, 54 subgingival plaque samples, 27 from healthy and 27 from periodontitis individuals, were used. The studies that were initially considered to be included in the analysis, along with the basis of exclusion of the excluded studies can be found in Supplementary Table S1. The workflow of our meta-analysis is presented in Figure 1.

**Table 1:** Sampling details of each study included in the meta-analysis.

Study	Geographical origin	Sample inclusion and definition of periodontitis	Number of samples	
			Healthy	Periodontitis
Duran-Pinedo et al. (2014)	USA	All healthy and periodontitis samples. Healthy sites had PD < 3 mm while periodontitis sites had PD > 5 mm and CAL > 3 mm	6	7
Jorth et al. (2014)	Turkey	All healthy and periodontitis samples. Periodontitis sites had PD ≥ 5 mm and CAL ≥ 5 mm	3	3
Yost et al. (2015)	USA	Samples from stable tooth sites from first visit (0 months) were defined as healthy and samples from progressive site from last visit (2 months) were defined as periodontitis (PD ≥ 4.0 mm and CAL ≥ 4 mm)	8	8
Belstrøm et al. (2021)	Denmark	All healthy and periodontitis samples. Periodontitis sites displayed PD ≥ 6 mm and CAL ≥ 4 mm	10	9

PD, pocket depth; CAL, clinical attachment loss.

### 3.2 Microbial diversity (alpha-and beta-diversity)

**Figure 2:**
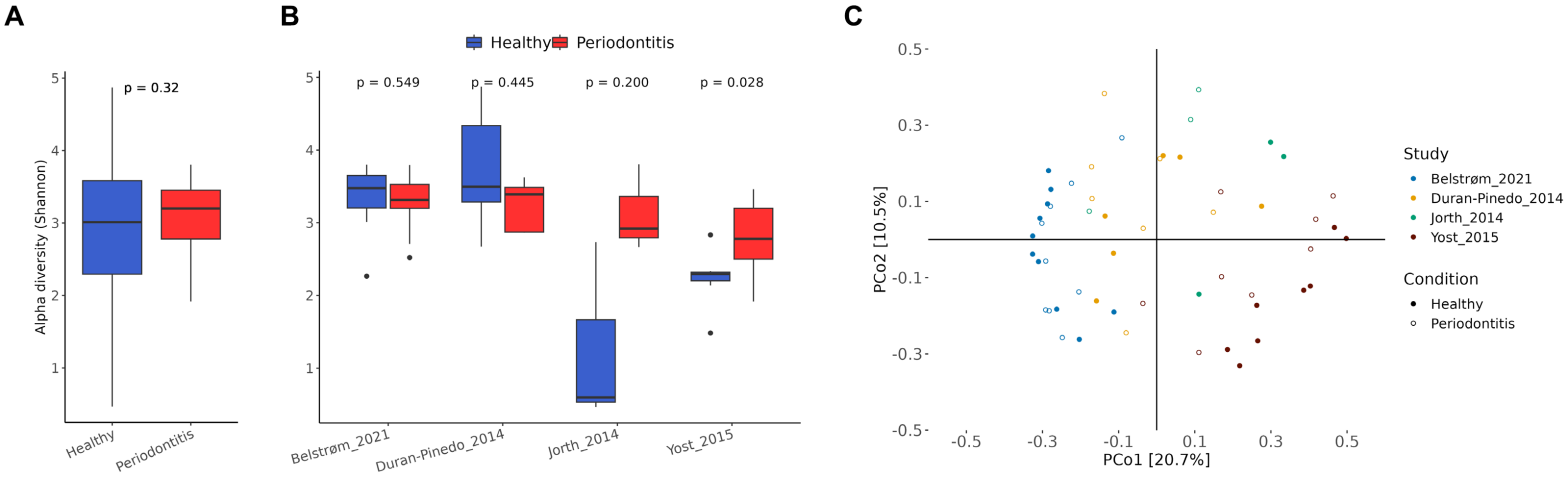
Microbial species diversity. **(A)** Box plot of α-diversity (Shannon index) in healthy (blue) and periodontitis (red) groups of all studies combined. For statistical analysis, blocked Wilcoxon test was used to treat “study” as a blocking factor. The *p*-value is shown on the top of the figure. **(B)** Box plot of α-diversity in healthy (blue) and periodontitis (red) groups in each individual study. The *p*-values of the two-sided Wilcoxon tests are shown at the top of the figures. **(C)** Principal coordinate analysis (PCoA) with Bray–Curtis dissimilarity matrix of the CSS normalized species abundances.

After preprocessing of the raw RNA-seq data, the abundance of microbial species was determined by AGAMEMNON (Skoufos et al., 2022) by using an extensive database containing bacterial, archaeal, viral and fungal reference genomes. The microbial α-diversity, as measured by Shannon index, was not significantly different (*p*-value > 0.05) between the healthy and periodontitis study groups (Figure 2A). This was also the case in each individual study of the meta-analysis, except for Yost et al. (2015), where the periodontitis group displayed greater α-diversity than the healthy group (Figure 2B).

The PCoA using Bray–Curtis dissimilarity matrix on CSS normalized species abundance data, can be seen in Figure 2C. The samples on the PCoA tended to cluster together based on their study origin rather than their dental health status, suggesting a significant impact of the “study” factor on species composition. To further investigate the effect of the confounding factors, the proportion of the total variance that can be attributed to each confounding factor (study, normalized library size, smoking status) and the variance explained by dental health status was quantified (Supplementary Figure S1). The analysis showed that the “study” factor had the strongest effect on the microbial species composition. This was expected, since the studies varied in both biological and technical aspects.

### 3.3 Differentially abundant species and genera between healthy and periodontitis

**Figure 3:**
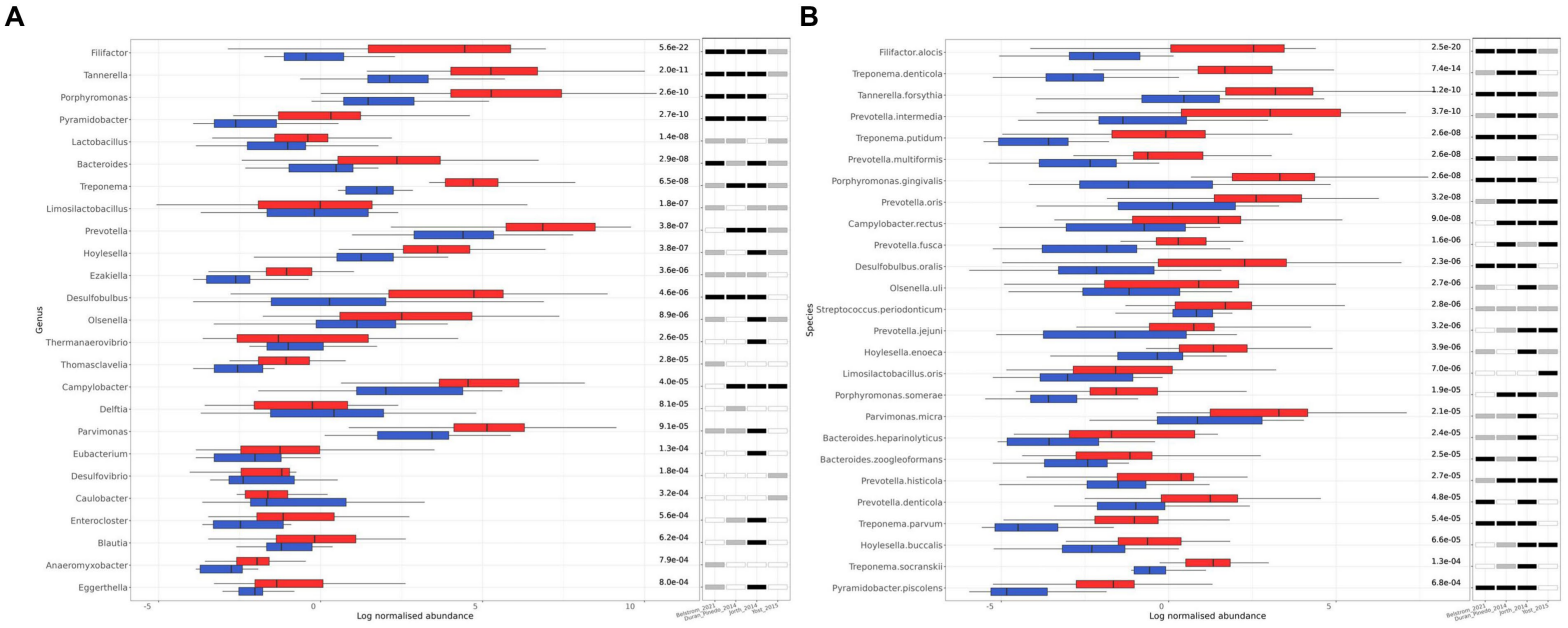
Differentially abundant genera and species between healthy (blue) and periodontitis (red) groups identified by MaAsLin2. **(A)** The 25 differentially abundant genera (FDR ≤ 10^−3^). **(B)** The 26 differentially abundant species (FDR ≤ 10^−3^). The log of CSS normalized abundance is presented. The FDR values are shown on the right side of the bar plot. The statistical significance in each individual study included in the meta-analysis is also shown. A white fill indicates an FDR > 0.05, a grey fill indicates an FDR ≤ 0.05, and a black fill indicates FDR ≤ 10^−3^.

The microbial genera that were differentially abundant (FDR ≤ 10^−3^) between healthy and periodontitis were 25, all of which were bacterial (Figure 3A). The only genera that were more abundant in the healthy group were *Delftia* and *Caulobacter,* while the remaining 23 were increased in periodontitis, including *Filifactor*, *Tannerella*, *Porphyromonas*, *Lactobacillus*, *Bacteroides*, *Treponema*, *Limosilactobacillus*, *Prevotella*, and *Hoylesella,* which exhibited the lowest calculated FDR values, in ascending order. To find out the number of individual studies in which each identified genus is differentially abundant, we performed a similar analysis for each study of the meta-analysis, separately. For the genera with the lowest FDR values, statistical significance was found in at least three of the studies (Figure 3A).

On the species level, 26 species were found to be differentially abundant (FDR ≤ 10^−3^), all of which were bacteria, and they were more abundant in the periodontitis group (Figure 3B). A more extensive list of the differentially abundant microbial species with an FDR ≤ 0.05 can be found in Supplementary Table S2. The top 10 species with the lowest FDR, in ascending order, were the following: *F. alocis, T. denticola, T. forsythia, P. intermedia, T. putidum, P. multiformis, P. gingivalis, P. oris, C. rectus, D. oralis.* All the identified bacterial species had very high prevalence among the samples (>50%) and have been previously associated with oral microbiome or periodontitis (Curtis et al., 2000; Teles et al., 2000; Abusleme et al., 2013; Pérez-Chaparro et al., 2014; Lasserre et al., 2018; Huang et al., 2021). The differentially abundant species were also found to be differentially abundant in most of the individual studies (Figure 3B).

### 3.4 Core microbiota

The core microbiota for each dental health condition was also identified. This was defined with species that had at least an 80% occurrence within the dental health group and their in-sample relative abundance was among the top 25% of the samples. The number of identified microbial species in the core microbiota of healthy and periodontitis groups were 40 and 80, respectively (Supplementary Table S3), meaning that the periodontitis group had a richer core microbiota. The number of shared species between the healthy and periodontitis core microbiota was 38, as only 2 bacterial species were exclusively found in the healthy core, namely, *L. mirabilis* and *N. sicca.* The number of species that were part of the core microbiota of either healthy and/or periodontitis and were found to be enriched in the periodontitis group were 18 (out of the 26 differentially abundant) (Table 2). Four species, *T. forsythia, P. gingivalis*, *S. periodonticum*, and *P. micra,* were found in both core microbiota, while the remaining 14, only in the periodontitis core.

**Table 2:** Bacterial species that are part of the core microbiota of healthy and/or periodontitis group, which have been found to be overrepresented in periodontitis.

Species	Mean relative abundance %		FDR
	Healthy	Periodontitis	
*Filifactor alocis*	4.78 × 10^−3^	6.93 × 10^−1^	2.51 × 10^−20^
*Treponema denticola*	9.28 × 10^−3^	5.95 × 10^−1^	7.40 × 10^−14^
***Tannerella forsythia***	1.76 × 10^−1^	3.90	1.23 × 10^−10^
*Prevotella intermedia*	5.62 × 10^−2^	2.22	3.72 × 10^−10^
*Treponema putidum*	7.35 × 10^−4^	1.14 × 10^−1^	2.55 × 10^−8^
***Porphyromonas gingivalis***	1.49 × 10^−1^	3.30	2.62 × 10^−8^
*Prevotella oris*	1.70 × 10^−1^	1.02	3.25 × 10^−8^
*Campylobacter rectus*	3.75 × 10^−2^	3.27 × 10^−1^	8.97 × 10^−8^
*Desulfobulbus oralis*	1.54 × 10^−1^	1.78	2.34 × 10^−6^
*Olsenella uli*	3.28 × 10^−2^	4.26 × 10^−1^	2.71 × 10^−6^
***Streptococcus periodonticum***	5.89 × 10^−2^	7.37 × 10^−1^	2.84 × 10^−6^
*Prevotella jejuni*	3.96 × 10^−2^	4.44 × 10^−1^	3.19 × 10^−6^
*Hoylesella enoeca*	4.63 × 10^−2^	3.05 × 10^−1^	3.89 × 10^−6^
***Parvimonas micra***	2.70 × 10^−1^	2.36	2.10 × 10^−5^
*Prevotella histicola*	1.54 × 10^−2^	5.05 × 10^−2^	2.75 × 10^−5^
*Prevotella denticola*	5.46 × 10^−2^	1.23	4.77 × 10^−5^
*Hoylesella buccalis*	7.51 × 10^−3^	2.38 × 10^−2^	6.56 × 10^−5^
*Treponema socranskii*	5.66 × 10^−2^	1.51 × 10^−1^	1.30 × 10^−4^

Species in bold were part of the core microbiota of both health status groups, while the rest of the species were only part of the periodontitis core microbiota. The mean relative abundance % of the species in each health group, and the FDR is also shown.

### 3.5 Differentially transcribed gene families (UniRef90) and virulence factors

**Figure 4:**
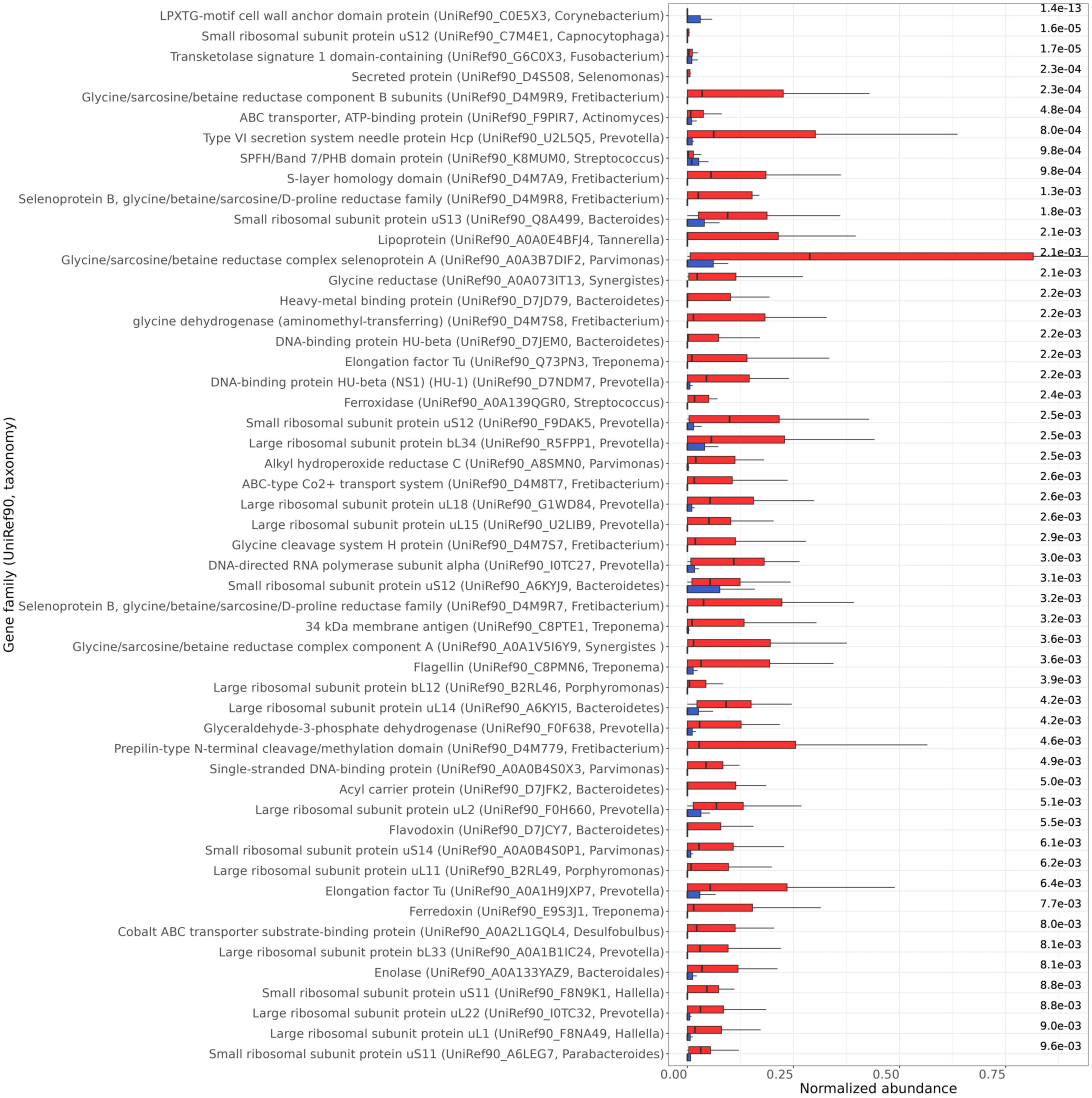
Differentially transcribed gene families (UniRef90, taxonomy) between healthy (blue) and periodontitis (red) health groups identified by MaAsLin2. The normalized abundance of the 52 gene families, with FDR ≤ 10^−2^, are presented. The FDR are shown on the right side of the bar plot.

Functional analysis showed that 52 gene families (UniRef90) were differentially transcribed between the healthy and periodontitis groups, with an FDR ≤ 10^−2^ and a total sample prevalence of at least 25% (Figure 4). The uncharacterized proteins that were found to be differentially transcribed are not presented, but they can be found in the Supplementary Table S4. Fifty of the differentially transcribed gene families were more abundant in periodontitis and they are involved in transmembrane transport and secretion, amino acid metabolism, surface protein and flagella synthesis, energy metabolism, and DNA supercoiling. These genes are expressed by bacterial genera that are known to be involved in periodontitis such as *Capnocytophaga, Fusobacterium, Porphyromonas, Fretibacterium, Prevotella, Streptococcus, Synergistes, Tannerella, Parvimonas, Treponema*, and *Desulfobulbus* (Huang et al., 2021). Only two gene families, encoding the LPXTG-motif cell wall anchor domain protein (UniRef90_C0E5X3) from *Corynebacterium* and the PHB domain protein (UniRef90_K8MUM0) from *Streptococcus*, were found to be more abundant in healthy samples (Figure 4). Interestingly, statistical significance (FDR ≤ 0.05) of the differential transcription of the identified gene families was not achieved in each study separately (except from elongation factor Tu—UniRef90_Q73PN3 and 34 kDa membrane antigen—UniRef90_C8PTE1 in Jorth et al., 2014), further highlighting the importance of meta-analysis for the identification of gene families with an important role in periodontitis.

**Figure 5:**
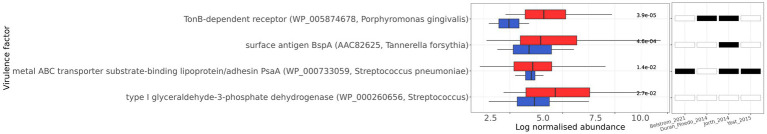
Differentially transcribed virulence factors between healthy (blue) and periodontitis (red) health groups. The log of normalized abundance of the 4 virulence factor genes with FDR ≤ 0.05 are presented. The name of the genes and the NCBI accession (in parenthesis) are shown. The FDR are shown on the right side of the bar plot. The statistical significance in each individual study included in the meta-analysis is also shown. White fill indicates FDR > 0.05, while black fill indicates FDR ≤ 0.05.

For the identification of bacterial virulence factor genes, the virulence factor genes database (VFDB) was used. Four virulence factor genes were found to be differentially transcribed (FDR ≤ 0.05) in periodontitis (Figure 5), and they originate from known periodontal pathogens. In particular, these virulence factors were the TonB-dependent receptor from *P. gingivalis*, BspA from *T. forsythia*, and two from the *Streptococcus* genus: metal ABC transporter substrate-binding lipoprotein/adhesin PsaA, and the Type I glyceraldehyde-3-phosphate dehydrogenase (GAPDH).

### 3.6 Co-occurrence networks of microbial species

**Figure 6:**
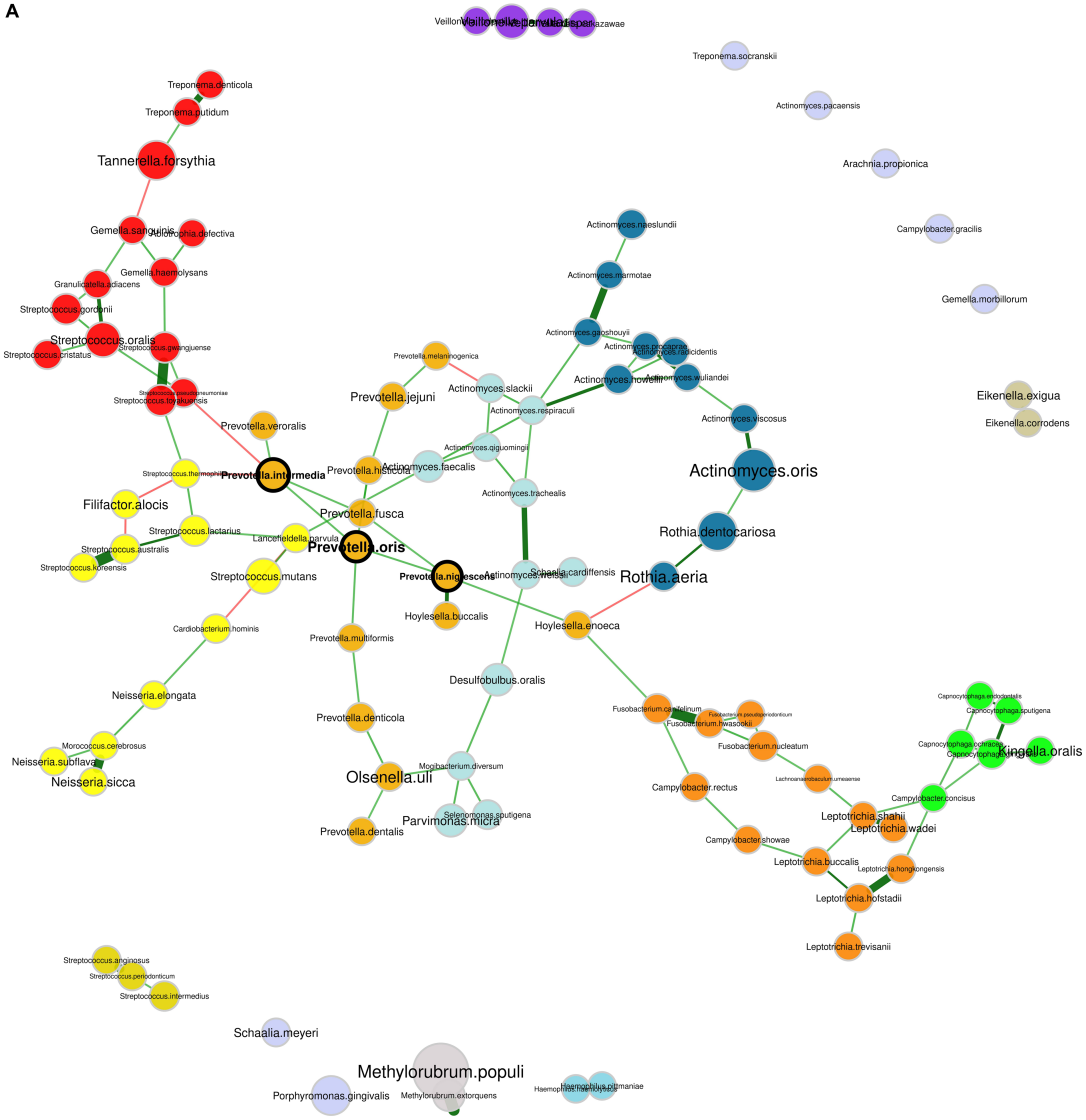

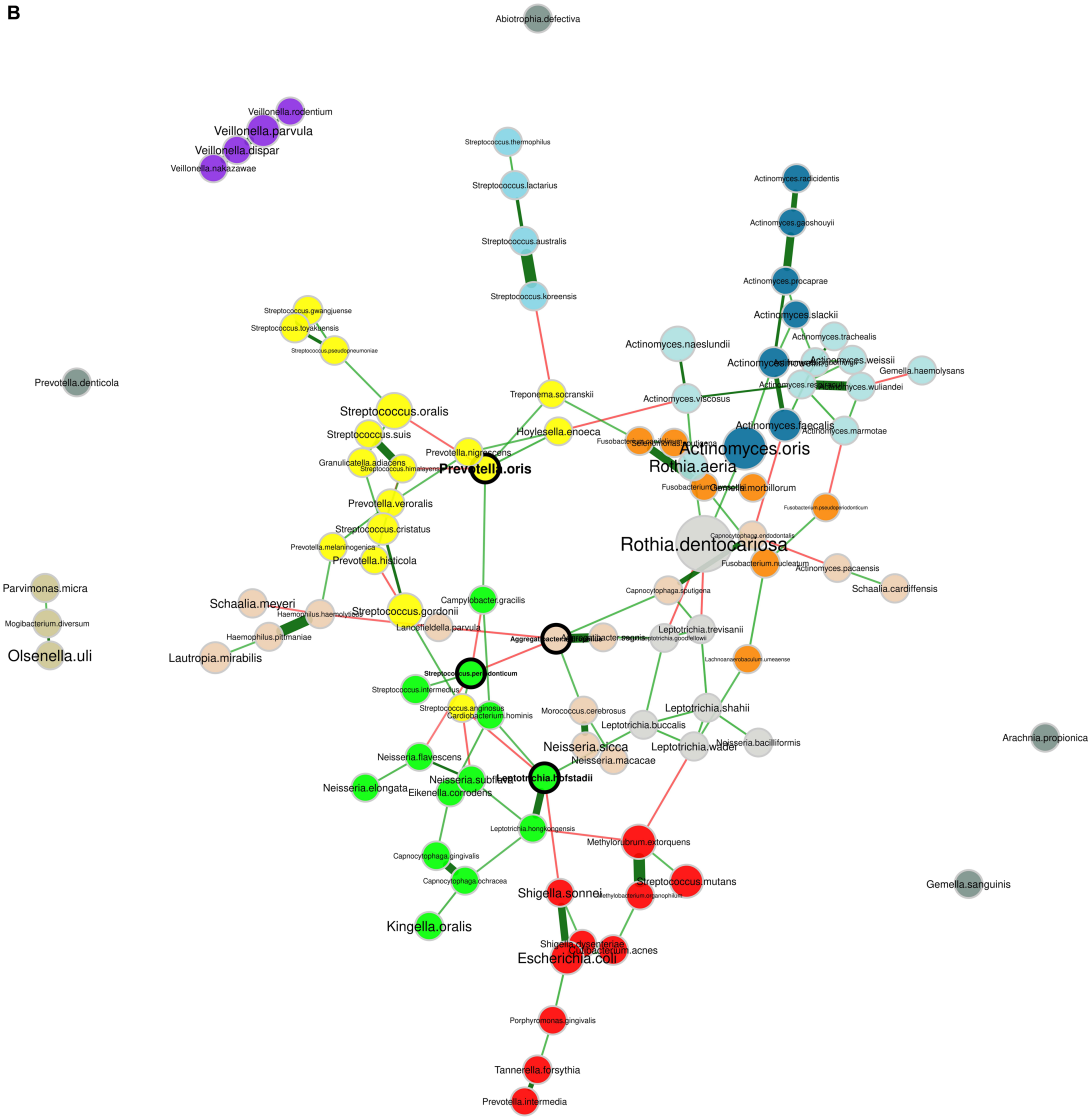
Bacterial co-occurrence networks. **(A)** Network of the subgingival microbiome in periodontitis. **(B)** Network of the healthy subgingival microbiome. The nodes represent the bacterial species and their size is scaled according to the sum of normalized Cumulative Sum Scaling (CSS) values, specific to each bacterial species. Species that are considered hubs are marked in bold and species from different clusters, as determined by the fast greedy algorithm, are marked in different colors. Positive and negative correlations are depicted with green and red edges, respectively, while the thickness of the edges corresponds to the strength of the correlation.

The microbial network analysis of healthy individuals and patients with periodontitis uncovered different “hub” species (Figure 6). Hub species are defined as species that are highly connected with others, and they are considered critical for a network’s structure. In the healthy group, the identified hub microorganisms were *Aggregatibacter aphrophilus*, *Leptotrichia hofstadii*, *Prevotella oris*, and *Streptococcus periodonticum*. In the periodontitis group, the hubs were species that belonged to the *Prevotella* genus, namely, *P. intermedia*, *P. nigrescens* and *P. oris*. *P. intermedia*, and *P. oris* were found to be significantly more abundant in periodontitis, highlighting a potentially important role of these organisms in the overall network’s shift toward the periodontitis state. Supplementary Table S5 shows the topological parameters of the networks in the healthy and periodontitis state. The subgingival microbiome of the periodontitis subjects constituted a less dense bacterial network compared to healthy controls. Specifically, in the periodontitis network, the overall edge density was equal to 0.021 as compared to 0.025 in the healthy network. However, the positive edge percentage, reflecting the co-existence of bacterial species, was 81.45% in healthy individuals and 92.52% in periodontitis patients, suggesting a higher level of positive interaction between microbial species in the periodontal disease state.

## 4 Discussion

In our meta-analysis, we observed a higher number of microbial species in the core microbiota of the periodontitis group compared to the healthy group, with the former exhibiting twice the number of species (80 versus 40, respectively). Consistent with findings from Abusleme and co-authors, an increased number of core species in the periodontitis group can be attributed to the emergence of new species during the development of periodontitis, without the replacement of primary health-associated species (Abusleme et al., 2013). In the healthy state, the Gram-positive bacteria *Actinomyces*, *Streptococcus*, *Rothia*, and *Gemella,* and to a lesser extent, the Gram-negative bacteria *Veillonella, Neisseria, Leptotrichia*, *Fusobacterium*, and *Lautropia* dominated the dental microbiota. However, under disease conditions, there was a notable shift toward Gram-negative bacteria with a pathogenic role such as, *Tannerella*, *Prevotella*, *Porphyromonas*, *Treponema*, *Campylobacter*, *Pyramidobacter, Bacteroides*, and *Desulfobulbus*, which aligns closely with findings from Ezzo and Cutler (2000). Other bacterial genera with eminent presence in periodontal disease included *Capnocytophaga*, *Fusobacterium*, *Filifactor*, *Leptotrichia*, and *Hoylesella.*

As expected, prominent periodontal pathogens, including *F. alocis*, *T. forsythia*, *P. gingivalis*, *T. denticola*, *P. intermedia, C. rectus, D. oralis*, and *S. periodonticum* were significantly more abundant in periodontitis. However, none of these species was differentially abundant in all individual studies with an FDR threshold of 10^−3^, while statistical significance (FDR ≤ 0.05) was achieved only for seven of these species across all studies. This highlights the importance of meta-analysis for the identification of microbial species with important role in periodontitis.

The functional analysis identified 50 gene families (UniRef90) with significantly increased transcription levels in the periodontitis group. Most of these gene families originate from bacterial genera with known periodontal pathogen members, and they are involved in various processes of bacterial metabolism. In particular, ABC transporters from *Actinomyces* and *Fretibacterium*, and metal ABC transporters from *Desulfobulbus* (cobalt) and *Streptococcus* (manganese) displayed higher transcription levels in periodontitis. Increased transcription levels of ABC transporters in periodontitis have been previously reported (Duran-Pinedo et al., 2014; Szafrański et al., 2015; Yost et al., 2015). Besides their role as transporters, these proteins may also function as adhesins, for the attachment to the host cells, as shown for the virulence factor PsaA, which is part of the ABC-type Mn^+2^ transport protein complex of *S. pneumoniae* (Rajam et al., 2008). A number of surface proteins, with known virulence functions, were also found to be enriched in periodontitis. Increased levels of TonB-dependent receptors, which are involved in hemin and hemoglobin utilization (Dashper et al., 2000; Simpson et al., 2000), were identified for *P. gingivalis*, congruent with Yost et al. (2015) and Szafrański et al. (2015). Higher transcription of prepilin from *Fretibacterium* was detected in periodontitis. Pili (or fimbriae) are filamentous structures on the bacterial cell surface, with crucial role in virulence and host cell attachment (Yoshimura et al., 2009; Ellison et al., 2022). The pathogenic role of type IV pilins has been reported for the periodontitis associated bacteria *P. gingivalis* (Choi et al., 2012)and *Eikenella corrodens* (Hood and Hirschberg, 1995), and their enhanced expression under periodontal disease conditions has been previously reported (Szafrański et al., 2015). Transcription of flagellin from *Treponema* was also enhanced in periodontal disease. The flagella confer the characteristic spiral morphology of spirochetes and play an important role in their motility and host tissue infection (Kurniyati et al., 2023), and their up-regulation in disease has been reported (Duran-Pinedo et al., 2014; Szafrański et al., 2015; Yost et al., 2015). Other up-regulated surface proteins with a potential pathogenic role, identified in the periodontitis state, were the BspA of *T. forsynthia* (Sharma et al., 1998; Mahalakshmi et al., 2018), the s-layer homology protein of *Fretibacterium*, and a lipoprotein cluster of *Tannerella. Prevotella* is known to secrete proteins in both planktonic and biofilm lifestyles (Karched et al., 2022) and the increased transcription of the type VI secretion system protein Hcp suggests an important role of the *Prevotella* secretome in periodontitis. Elongation factor thermal unstable (EF-Tu) is one of the most abundant proteins in bacteria, with an important role in protein synthesis. However, these proteins could also display “moonlighting” functions on the surface of the bacterial cells, where they can operate as adhesins and stimulate immune response (Harvey et al., 2019). EF-Tu proteins from *Treponema* and *Prevotella* were up-regulated, indicating that these proteins may contribute to the pathogenicity of *Treponema* and *Prevotella* in periodontitis.

Periodontal pockets are known to contain high levels of peptides and amino acids which emanate from the degradation of host serum or damaged tissue, by the proteolytic activity of the microbial communities and the immune response (Teles et al., 2000). A number of differentially transcribed genes under the periodontal disease state are involved in glycine metabolism. More specifically, glycine reductases from bacterial genera *Fretibacterium*, *Synergistes*, and *Parvimonas,* and glycine cleavage system H-and T-proteins from the *Fretibacterium* genus, exhibited increased transcription. Glycine reductases are involved in metabolic pathways that lead to energy production from glycine catabolism. Glycine reductases consist of 3 protein fractions, A and B selenoproteins and fraction C, and they partake in ATP production through the conversion of glycine to acetyl phosphate by using inorganic phosphate and the reducing potential of thioredoxin (Andreesen, 2004). In line with our results, the growth of the periodontal pathogen *T. denticola* has been reported to be impaired by the inhibition of selenium metabolism (Jackson-Rosario and Self, 2009). The glycine cleavage system comprises 4 proteins called T-, P-, L-and H-protein, and catalyzes the formation of serine from glycine (Szafrański et al., 2015). Serine is converted to pyruvate by the serine dehydratase, which can then be further metabolized by the ferredoxin oxidoreductase, or by the flavodoxin oxidoreductase under iron-limiting conditions (Buckel and Thauer, 2018). Ferredoxin from *Treponema* and flavodoxin from *Bacteroidetes,* were also enriched in periodontitis, in agreement with Szafrański et al. (2015). Interestingly, glycine has been shown to have an anti-inflammatory effect in gingival epithelial cells (Schaumann et al., 2013). Overall, the results indicate the important role of glycine catabolism and energy metabolism rewiring, for subgingival survival and for periodontal disease development.

Enhanced transcription of the DNA-binding protein HU from *Prevotella* and *Bacteroidetes* was identified under the periodontal disease state. HU has been reported to affect the transcription of surface polysaccharides in *P. gingivalis* (Alberti-Segui et al., 2010), and therefore, our results connote that this DNA-binding protein might function as a global regulator of genes involved in periodontitis.

The study of microbial co-occurrence networks could aid us in unveiling the complex interactions of microbial communities in dental plaque. Understanding the ecological relationships among bacterial species can guide mitigation strategies targeting critical bacterial interactions. In the present study, the network of healthy individuals, concordant with previous studies (Relvas et al., 2021; Zhang et al., 2022), was more interconnected but comprised fewer positive interconnections between its members compared to the periodontitis network. This implies that synergistic interactions among oral bacteria could play a significant role in shaping the microbial community associated with periodontitis. Furthermore, a notable shift in the interconnections was evident for three prominent pathogenic bacteria, namely *T. denticola*, *T. forsythia*, and *P. gingivalis*, as the transition from a healthy state to periodontitis occurred. This observation supports the notion that periodontitis represents a perturbation affecting the entire bacterial network, rather than being primarily driven by the antagonism among oral bacteria.

Socransky and co-authors, by using DNA probes, were able to quantify and cluster 40 bacterial species in microbial color complexes, based on their co-occurence (Socransky et al., 1998). Intriguingly, the clusters identified in our periodontitis network exhibited similarities with the microbial complexes reported in their study. In particular, *T. forsythia*, *T. denticola*, and *T. putidum* belonged to the same cluster resembling the first (red) complex containing *T. forsythia* (then *B. forsythus*), *T. denticola*, and *P. gingivalis*; *Prevotella* species, including *P. intermedia*, *P. nigrescens*, and *P. oris*, were positively correlated with *Fusobacterium* (e.g., *F. nucleatum* and *F. periodonticum*) and *Campylobacter* (e.g., *C. rectus* and *C. showae*) species taking after the second (orange) complex. Lastly, all *Streptococcus* species were positively correlated sharing similarities with the third (yellow) complex, while the cluster containing all the *Veillonella* species resembled the fifth (purple) complex. The positive correlation of these bacterial species might either signify the shared competence of these species for specific environmental conditions, or it might imply a genuine ecological cooperation through metabolite exchanges (Faust et al., 2012; Relvas et al., 2021).

Metatranscriptomics excludes the genomic material of dead and inactive cells and could generate more relevant information on the role of microbes in human health (Hempel et al., 2022; Ojala et al., 2023). By utilizing four distinct metatranscriptome datasets, our meta-analysis has produced robust and reliable results, pinpointing signature microbial species, gene families, virulence factor genes, and microbial relationships crucial in periodontal disease. The bacterial species and gene families identified as pivotal in the context of periodontitis, may serve as focal points for future strategies aimed at disease mitigation and drug development. To further advance our understanding of periodontal disease and its progression, longitudinal studies in conjunction with multiomics studies combining transcriptomics, proteomics, and metabolomics, could be used to associate genes, functional proteins and metabolites involved in periodontitis (Huang et al., 2021). Additionally, host-pathogen interaction studies could elucidate the interplay between the microbial community and the host and its immune response (Cugini et al., 2021).

## Data availability statement

The original contributions presented in the study are publicly available. This data can be found here: https://github.com/DianaLabUTH/Oral_Metatranscriptome_MetaAnalysis. Bioinformatic open-source tools and parameters used in this present study are defined in the “Methods” section and scripts can be found in the following Github repository: https://github.com/dianalabgr/Oral_Metatranscriptome_MetaAnalysis.

## Author contributions

AO: Conceptualization, Formal analysis, Methodology, Software, Visualization, Writing – original draft, Writing – review & editing. FK: Conceptualization, Methodology, Project administration, Writing – review & editing. AS: Conceptualization, Methodology, Software, Visualization, Writing – original draft, Writing – review & editing. AH: Conceptualization, Funding acquisition, Methodology, Project administration, Supervision, Writing – review & editing.
