# Supplementary material for: Microbial signatures in human periodontal disease: a metatranscriptome meta-analysis
Source: Front Microbiol. 2024 Apr 9;15:1383404. doi: 10.3389/fmicb.2024.1383404 (PMC11041396; doi:10.3389/fmicb.2024.1383404)
Supplement: Supplementary file 1 [file Data_Sheet_1.zip › Supplementary_Figure_S1.docx]

**A**


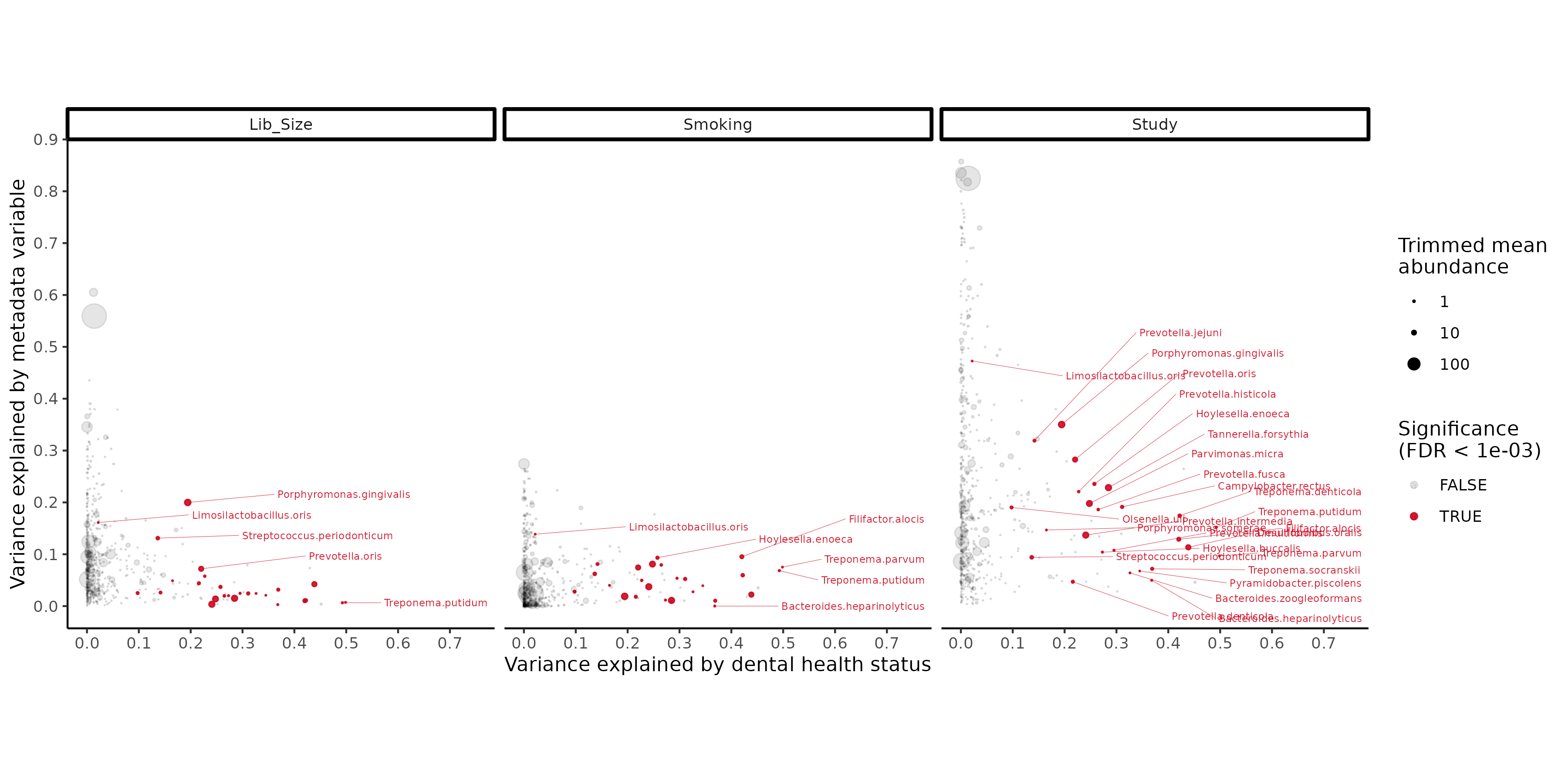


**B**


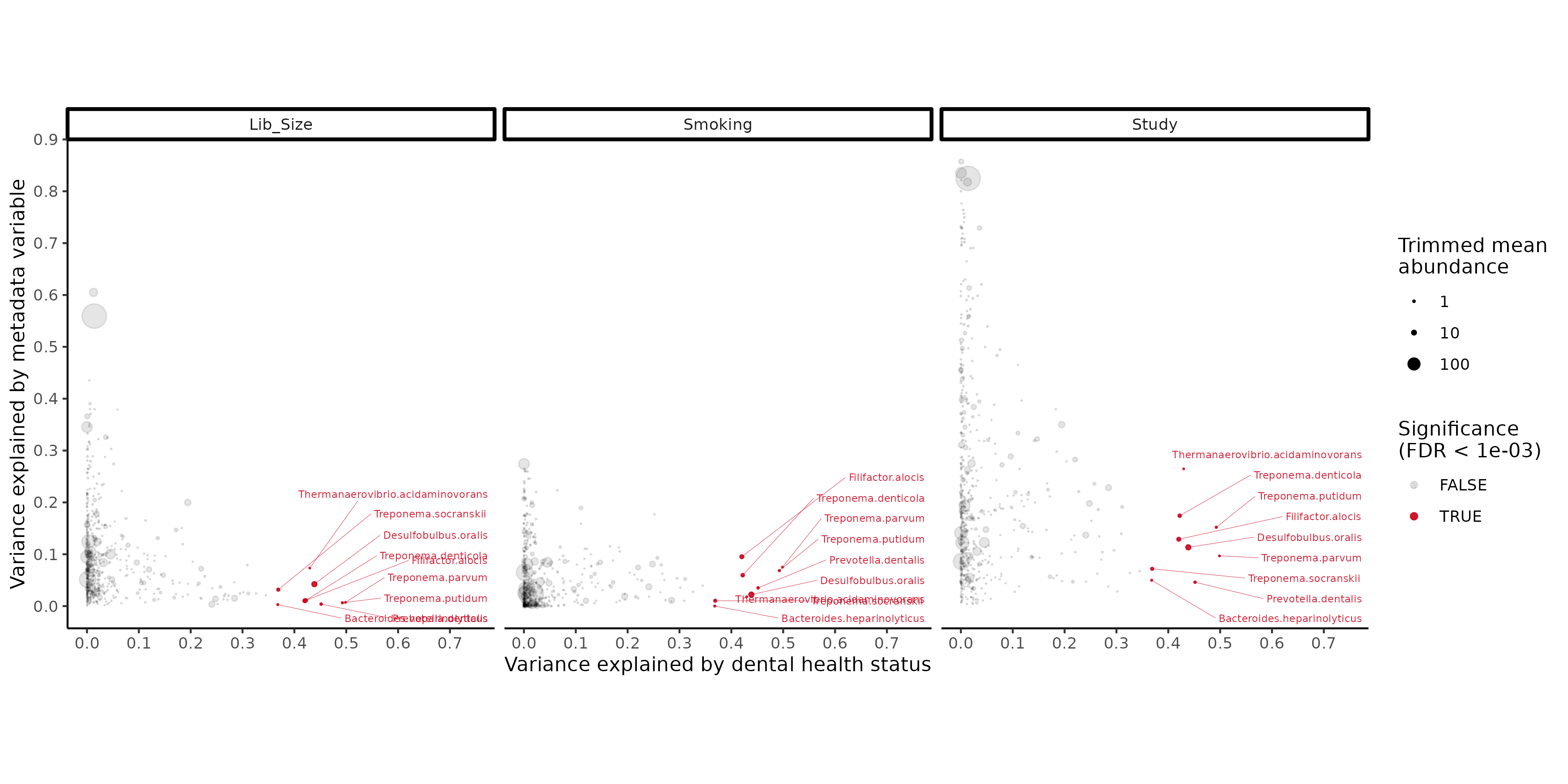


**FIGURE S1**. The variance explained by the confounding factors (normalized library sizes, smoking status and study origin) against the variance explained by the oral health status, with (**A**) and without (**B**) setting the study factor as a random effect. The analysis of the confounding factors for species abundance was conducted following the methodology described by Wirbel et al., 2019. Briefly, the total variance within the abundance of a given microbial species was compared to the variance explained by disease status and the variance explained by the confounding factor, with a linear model assumption. Variance calculations were performed with a non-parametric approach to account for non-Gaussian distribution of microbiome abundance data. The library size potential confounder, with continuous values, was transformed into categorical data as quartiles. The size of the spots represents the trimmed mean abundance. The species that have been found to have statistically significant abundance (qval ≤ 10-3), after differential analysis with MaAsLin2, are shown in red.

**References**

1. Wirbel, J., Pyl, P. T., Kartal, E., Zych, K., Kashani, A., Milanese, A., Fleck, J. S., Voigt, A. Y., Palleja, A., Ponnudurai, R., Sunagawa, S., Coelho, L. P., Schrotz-King, P., Vogtmann, E., Habermann, N., Niméus, E., Thomas, A. M., Manghi, P., Gandini, S., … Zeller, G. (2019). Meta-analysis of fecal metagenomes reveals global microbial signatures that are specific for colorectal cancer. *Nature Medicine*, *25*(4), 679–689. <https://doi.org/10.1038/s41591-019-0406-6>
