# Supplementary material for: Microbial signatures in human periodontal disease: a metatranscriptome meta-analysis
Source: Front Microbiol. 2024 Apr 9;15:1383404. doi: 10.3389/fmicb.2024.1383404 (PMC11041396; doi:10.3389/fmicb.2024.1383404)

**TABLE S1**. Studies that were considered for our meta-analysis. The study details, the decision on inclusion outcome (YES/NO) and the justification of exclusion are shown.

| **A/A** | **Authors** | **Title** | **Year** | **Journal** | **DOI** | **Inclusion (YES/NO)** | **Reason of Exclusion** |
| --- | --- | --- | --- | --- | --- | --- | --- |
| 1 | Zhong et al. | Metagenome and Metatranscriptome Analyses Using Protein Family Profiles. | 2016 | PLoS Comput. Biol. | 10.1371/journal.pcbi.1004991 | NO | A homology detection algorithm is presented. New sequencing data was not generated. |
| 2 | Duran-Pinedo et al. | Subgingival host-microbiome metatranscriptomic changes following scaling and root planing in grade II/III periodontitis. | 2023 | J. Clin. Periodontol. | 10.1111/jcpe.13737 | NO | Stable and progressive dental sites are studied. No clearly healthy and periodontitis samples were produced. |
| 3 | Yost et al. | Potassium is a key signal in host-microbiome dysbiosis in periodontitis. | 2017 | PLoS Pathog. | 10.1371/journal.ppat.1006457 | NO | Same data used as in Yost et al., 2015. |
| 4 | Frias-Lopez & Duran-Pinedo | Effect of periodontal pathogens on the metatranscriptome of a healthy multispecies biofilm model. | 2012 | J. Bacteriol. | 10.1128/JB.06328-11 | NO | *In vitro* experimental data. |
| 5 | May et al. | metaModules identifies key functional subnetworks in microbiome-related disease. | 2016 | Bioinformatics | 10.1093/bioinformatics/btv526 | NO | MetaModules tool was introduced. No metatranscriptome data produced. |
| 6 | Belstrøm et al. | Transcriptional Activity of Predominant *Streptococcus* Species at Multiple Oral Sites Associate With Periodontal Status. | 2021 | Front. Cell Infect. Microbiol. | 10.3389/fcimb.2021.752664 | NO | No original metatranscriptome data produced. |
| 7 | Lewin et al. | A quantitative framework reveals traditional laboratory growth is a highly accurate model of human oral infection. | 2022 | Proc Natl Acad Sci U S A. | 10.1073/pnas.2116637119 | NO | No original metatranscriptome data produced. |
| 8 | Grischke et al. | Removable denture is a risk indicator for peri-implantitis and facilitates expansion of specific periodontopathogens: a cross-sectional study. | 2021 | BMC Oral Health | 10.1186/s12903-021-01529-9 | NO | Peri-implantitis was studied. |
| 9 | Aleti et al. | Identification of the Bacterial Biosynthetic Gene Clusters of the Oral Microbiome Illuminates the Unexplored Social Language of Bacteria during Health and Disease. | 2019 | mBio | 10.1128/mBio.00321-19 | NO | No metatranscriptome data produced. |
| 10 | Yost & Duran-Pinedo | The contribution of Tannerella forsythia dipeptidyl aminopeptidase IV in the breakdown of collagen. | 2018 | ΜοΙ. Oral. Microbiol. | 10.1111/omi.12244 | NO | No metatranscriptome data was produced. |
| 11 | Jorth et al. | Metatranscriptomics of the Human Oral Microbiome during Health and Disease. | 2014 | mBio | 10.1128/mBio.01012-14 | YES |  |
| 12 | Belstrøm et al. | Periodontitis associates with species-specific gene expression of the oral microbiota. | 2021 | NPJ Biofilms and Microbiomes | 10.1038/s41522-021-00247-y | YES |  |
| 13 | Nowicki et al. | Microbiota and Metatranscriptome Changes Accompanying the Onset of Gingivitis. | 2018 | mBio | 10.1128/mBio.00575-18 | NO | The samples are from dental sites with gingivitis and not periodontitis. |
| 14 | Edlund et al. | Uncovering complex microbiome activities via metatranscriptomics during 24 hours of oral biofilm assembly and maturation. | 2018 | Microbiome | 10.1186/s40168-018-0591-4 | NO | *In vitro* oral biofilm used. Only first 24 h of biofilm development was studied. |
| 15 | Komatsu et al. | Discriminating Microbial Community Structure Between Peri-Implantitis and Periodontitis With Integrated Metagenomic, Metatranscriptomic, and Network Analysis. | 2020 | Frontiers in Cellular and Infection Microbiology | 10.3389/fcimb.2020.596490 | NO | Lack of healthy samples. Only periodontitis and peri-implantitis samples. |
| 16 | Duran-Pinedo et al. | Small RNA Transcriptome of the Oral Microbiome during Periodontitis Progression. | 2015 | AEM journal | 10.1128/AEM.01782-15 | NO | Same samples as in Duran-Pinedo et al., 2014 |
| 17 | Shiba et al. | Distinct interacting core taxa in co-occurrence networks enable discrimination of polymicrobial oral diseases with similar symptoms. | 2016 | Scientific Reports | 10.1038/srep30997 | NO | Lack of healthy samples. Only periodontitis and peri-implantitis samples. |
| 18 | Carda-Diéguez et al. | Functional changes in the oral microbiome after use of fluoride and arginine containing dentifrices: a metagenomic and metatranscriptomic study. | 2022 | Microbiome | 10.1186/s40168-022-01338-4 | NO | Study of fluoride-containing dentifrice on dental caries. Supragingival dental plaque samples |
| 19 | Duran-Pinedo et al. | Community-wide transcriptome of the oral microbiome in subjects with and without periodontitis. | 2014 | The ISME Journal | 10.1038/ismej.2014.23 | YES |  |
| 20 | Zhang et al. | Metatranscriptomic analysis of an in vitro biofilm model reveals strain-specific interactions among multiple bacterial species | 2019 | J. Oral Microbiol. | [10.1080/20002297.2019.1599670](https://doi.org/10.1080/20002297.2019.1599670) | No | An *in vitro* polymicrobial subgingival plaque model was used. |
| 21 | Yost et al. | Functional signatures of oral dysbiosis during periodontitis progression revealed by microbial metatranscriptome analysis. | 2015 | Genome Medicine | 10.1186/s13073-015-0153-3 | YES |  |
| 22 | Nemoto et al. | Discrimination of Bacterial Community Structures amongHealthy, Gingivitis, and Periodontitis Statuses through  Integrated Metatranscriptomic and Network Analyses. | 2021 | mSystems | 10.1128/msystems.00886-21 | NO | Raw data not available. |
| 23 | Szafrański et al. | Functional biomarkers for chronic periodontitis and insights  into the roles of Prevotella nigrescens and Fusobacterium  nucleatum; a metatranscriptome analysis. | 2015 | NPJ Bioﬁlms and Microbiomes | 10.1038/npjbioﬁlms.2015.17 | NO | Raw data not available. |
| 24 | Ram-Mohan & Meyer | Comparative Metatranscriptomics of Periodontitis Supports a Common Polymicrobial Shift in Metabolic Function and Identifies Novel Putative Disease-Associated ncRNAs | 2020 | Front. Microbiol. | 10.3389/fmicb.2020.00482 | No | No new sequencing data was produced. |
| 25 | Belstrøm et al. | Metagenomic and metatranscriptomic analysis of saliva reveals disease-associated microbiota in patients with periodontitis and dental caries | 2017 | NPJ Biofilms and Microbiomes | 10.1038/s41522-017-0031-4 | Νο | Saliva samples were used. |
| 26 | Beall et al. | Genomics of the Uncultivated, Periodontitis-Associated Bacterium *Tannerella* sp. BU045 (Oral Taxon 808) | 2018 | mSystems | 10.1128/mSystems.00018-18 | No | No metatranscriptome data was produced. |
| 27 | Joseph et al. | The Murine Oral Metatranscriptome Reveals Microbial and Host Signatures of Periodontal Disease | 2023 | [J. Dent. Res.](https://www.ncbi.nlm.nih.gov/pmc/articles/PMC10152569/) | 10.1177/00220345221149675 | No | No human samples. |
| 28 | Lewin et al. | A quantitative framework reveals traditional laboratory growth is a highly accurate model of human oral infection | 2022 | Proc. Natl Acad. Sci. USA |  | No | No new sequencing data was produced. |
| 29 | Shah et al. | The making of a miscreant: tobacco smoke and the creation of pathogen-rich biofilms | 2017 | NPJ Biofilms and Microbiomes | 10.1038/s41522-017-0033-2 | No | The article does not study periodontitis, but the effect of smoking |
| 30 | Edlund et al. | High-Quality Draft Genome Sequence of Low-pH-Active Veillonella parvula Strain SHI-1, Isolated from Human Saliva within an In Vitro Oral Biofilm Model | 2016 | Genome Announc. | 10.1128/genomeA.01684-15 | No | No metatranscriptome and subgingival samples. |
| 31 | Deng et al. | Worlds Apart – Transcriptome Profiles of Key Oral Microbes in the Periodontal Pocket Compared to Single Laboratory Culture Reflect Synergistic Interactions | 2018 | Front. Microbiol. | 10.3389/fmicb.2018.00124 | No | No metatranscriptome data was produced |
| 32 | Zou et al. | TrkA serves as a virulence modulator in *Porphyromonas gingivalis* by maintaining heme acquisition and pathogenesis | 2022 | Front. Cell. Infect. Microbiol. | [10.3389/fcimb.2022.1012316](https://doi.org/10.3389/fcimb.2022.1012316) | No | No metatranscriptome data was produced. |
| 33 | Fried et al. | Use of unbiased metagenomic and transcriptomic analyses to investigate the association between feline calicivirus and feline chronic gingivostomatitis in domestic cats | 2021 | Am. J. Vet. Res. | 10.2460/ajvr.82.5.381 | No | Human periodontitis was not studied. |
| 34 | Wang G.P. | Defining functional signatures of dysbiosis in periodontitis progression | 2015 | Genome Med. | 10.1186/s13073-015-0165-z | No | No sequencing data produced |
| 35 | Duran-Pinedo et al. | The effect of the stress hormone cortisol on the metatranscriptome of the oral microbiome | 2018 | NPJ Biofilms and Microbiomes | 10.1038/s41522-018-0068-z | No | Periodontitis was not studied. |
| 36 | Stashenko et al. | The Oral Mouse Microbiome Promotes Tumorigenesis in Oral Squamous Cell Carcinoma | 2019 | mSystems | [10.1128/mSystems.00323-19](https://doi.org/10.1128%2FmSystems.00323-19) | No | No human metatranscriptome data was produced. |
| 37 | Szafrański et al. | Quorum sensing of Streptococcus mutans is activated by Aggregatibacter actinomycetemcomitans and by the periodontal microbiome | 2017 | BMC Genomics | 10.1186/s12864-017-3618-5 | No | No periodontal metatranscriptome data was produced. Biofilms of of S. mutans, A. actinomycetemcomitans  were studied |
| 38 | Wattimena et al. | An in vitro model for studies of attenuation of antibiotic‐inhibited growth of Aggregatibacter actinomycetemcomitans Y4 by polyamines | 2021 | Mol. Oral Microbiol. | [10.1111/omi.12353](https://doi.org/10.1111%2Fomi.12353) | No | Periodontits was not studied and subgingival metatranscriptome data was not produced |


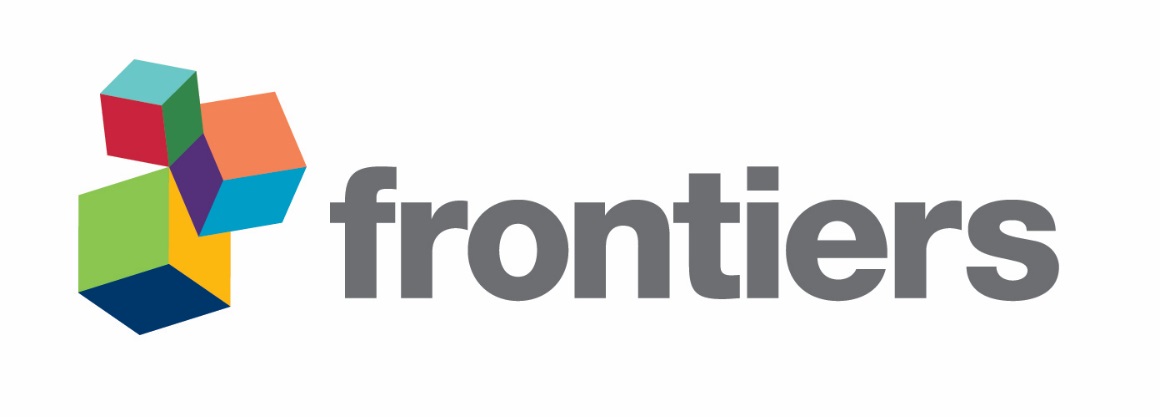

Supplement: Supplementary file 1 [file Data_Sheet_1.zip › Supplementary_Table_S1.docx]
