# Supplementary material for: Microbial signatures in human periodontal disease: a metatranscriptome meta-analysis
Source: Front Microbiol. 2024 Apr 9;15:1383404. doi: 10.3389/fmicb.2024.1383404 (PMC11041396; doi:10.3389/fmicb.2024.1383404)
Supplement: Supplementary file 1 [file Data_Sheet_1.zip › Supplementary_Table_S5.docx]

**Table S5**. Topological characteristics of co-occurrence networks of periodontitis and healthy individuals.

| **Whole network** | **Periodontitis** | **Healthy** |
| --- | --- | --- |
| Number of components | 13.00000 | 7.00000 |
| Clustering coefficient | 0.10170 | 0.13708 |
| Modularity | 0.75902 | 0.70086 |
| Positive edge percentage | 92.52336 | 81.45161 |
| Edge density | 0.02162 | 0.02505 |
| Natural connectivity | 0.01174 | 0.01187 |
